# Evaluation of a commercial SARS-CoV-2 multiplex PCR genotyping assay for variant identification in resource-scarce settings

**DOI:** 10.1371/journal.pone.0269071

**Published:** 2022-06-24

**Authors:** Chijioke N. Umunnakwe, Zinhle N. Makatini, Mathapelo Maphanga, Anele Mdunyelwa, Khamusi M. Mlambo, Puseletso Manyaka, Monique Nijhuis, Annemarie Wensing, Hugo A. Tempelman

**Affiliations:** 1 Ndlovu Research Centre and Laboratories, Dennilton, Limpopo Province, South Africa; 2 Ndlovu Research Consortium, Dennilton, Limpopo Province, South Africa; 3 Department of Virology, Faculty of Health Sciences, University of the Witwatersrand, Johannesburg, South Africa; 4 Department of Medical Microbiology, Virology, University Medical Center Utrecht (UMCU), Utrecht, The Netherlands; 5 HIV Pathogenesis Research Unit, Faculty of Health Sciences, University of the Witwatersrand, Johannesburg, South Africa; 6 Wits Reproductive Health and HIV Institute (Wits RHI), University of the Witwatersrand, Johannesburg, South Africa; CEA, FRANCE

## Abstract

The rapid emergence and spread of numerous severe acute respiratory syndrome coronavirus 2 (SARS-CoV-2) variants across the globe underscores the crucial need for continuous SARS-CoV-2 surveillance to ensure that potentially more pathogenic variants are detected early and contained. Whole genome sequencing (WGS) is currently the gold standard for COVID-19 surveillance; however, it remains cost-prohibitive and requires specialized technical skills. To increase surveillance capacity, especially in resource-scarce settings, supplementary methods that are cost- and time-effective are needed. Real-time multiplex PCR genotyping assays offer an economical and fast solution for screening circulating and emerging variants while simultaneously complementing existing WGS approaches. In this study we evaluated the Allplex^TM^ SARS-CoV-2 Variants II multiplex real-time PCR genotyping assay, Seegene (South Korea), and implemented it in retrospectively characterizing circulating SARS-CoV-2 variants in a rural South African setting between April and October 2021, prior to the emergence of the Omicron variant in South Africa. The Allplex^TM^ SARS-CoV-2 Variants II real-time PCR assay demonstrated perfect concordance with whole-genome sequencing in detecting Beta and Delta variants and exhibited high specificity, sensitivity and reproducibility. Implementation of the assay in characterization of SARS-CoV-2 variants between April and October 2021 in a rural South African setting revealed a rapid shift from the Beta to the Delta variant between April and June. All specimens successfully genotyped in April were Beta variants and the Delta variant was not detected until May. By June, 78% of samples genotyped were Delta variants and in July >95% of all genotyped samples were Delta variants. The Delta variant continued to predominate through to the end of our analysis in October 2021. Taken together, a commercial SARS-CoV-2 variant genotyping assay detected the rapid rate at which the Delta variant displaced the Beta variant in Limpopo, an under-monitored province in South Africa. Such assays provide a quick and cost-effective method of monitoring circulating variants and should be used to complement genomic sequencing for COVID-19 surveillance especially in resource-scarce settings.

## Introduction

Severe acute respiratory syndrome coronavirus 2 (SARS-CoV-2), the causative pathogen of coronavirus disease 2019 (COVID-19) was originally isolated from patient samples in Wuhan in December 2019 and has since spread worldwide resulting in the current global pandemic. SARS-CoV-2, like all viruses, generates random mutations during viral replication, resulting in genetic variation among circulating viral populations. Viral recombination, a process by which genetic material is exchanged between viral sequences, also contributes to genetic changes. Over time, such changes can lead to emergence of new variants that differ from the ancestral virus in transmissibility, virulence, immune escape, and/or disease presentation. Based on the public health impact, the World Health Organization (WHO) designates these variants as variants of concern (VOCs) or variants of interest (VOIs) [1].

As of January 2022, there were five VOCs, namely Alpha, Beta Gamma, Delta and Omicron and two VOIs, namely Lambda and Mu [1, 2]. All VOCs and VOIs contain mutations in the spike (S) gene, which may confer fitness advantages. The Alpha variant contains key deletions in S gene amino acid positions 69–70 and 144–145 in addition to other mutations in the S gene receptor binding domain (RBD). The Beta variant contains RBD mutations K417N and E484K in contrast to the Gamma variant, which contains K417T and E484K [1–5]. The Delta variant has RBD mutations L452R and P681R, and the Omicron variant contains a myriad of mutations in the Spike protein, some of which overlap with mutations found in preceding variants [6]. Reports indicate that the Delta and Omicron variants have higher transmissibility and cause more breakthrough infections and reinfections compared to the Alpha, Beta and Gamma variants [7, 8].

**Fig 1:**
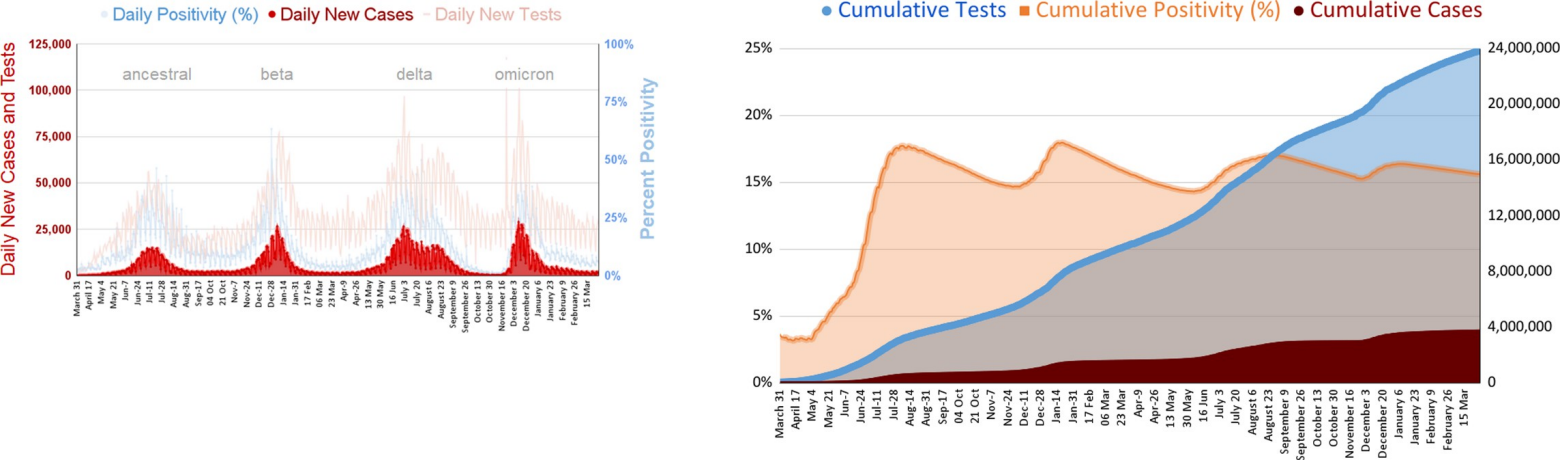
Daily and cumulative SARS-CoV-2 cases in South Africa, 2020–2022. (A) Daily infections and tests are shown on the left y-axis in dark and light red, respectively, and daily positivity is depicted on the right y-axis, in light blue. Predominant variants for each wave of infection are shown in grey font above the graphs. (B) Cumulative cases and tests are shown on the right y-axis in dark red and blue, respectively, and the cumulative positivity rate is depicted on the left y-axis in light orange. Data was retrieved from the National Institute of Communicable Diseases, Government of South Africa, National COVID-19 Daily Reports [9].

South Africa has experienced four waves of COVID-19 infections as of March 2022 (Fig 1A and 1B). The first wave occurred between March and September 2020, followed by a second wave, largely fuelled by the Beta variant, spanning from October 2020 to March 2021. The third wave, driven by the Delta variant, lasted from May till October 2021. More recently, the Omicron variant gave rise to the fourth wave, beginning in November and peaking in December 2021 (Fig 1A) [9–11]. The emergence and spread of these variants highlights the urgent need for surveillance of circulating SARS-CoV-2 sequences in the population to detect new mutations early. In South Africa, the Network for Genomics Surveillance (NGS-SA) led the country’s variant monitoring efforts using whole genome sequencing based approaches to identify the Beta, Delta and Omicron variants early [12–15].

Whole genome sequencing (WGS) remains a gold standard for virus surveillance and has been pivotal in tracking variants during the pandemic [4, 13, 14, 16]. However, WGS can be time-consuming, costly, and requires specialized technical skills. Furthermore, due to lack of adequate infrastructure in underdeveloped areas, this approach can be prone to sampling bias with urban and developed areas typically being over-represented among sequenced samples while rural populations remain under-sampled. For instance, in South Africa, the vast majority of sequenced samples are collected from urban and coastal provinces, which have better monitoring infrastructure, while the more rural provinces of South Africa remain severely underrepresented. To address these limitations, additional strategies that are cost-effective, rapid, and that do not require highly technical skills are needed. One strategy involves leveraging commercial SARS-CoV-2 real-time PCR genotyping assays that can quickly detect unique variant mutations without the need for expensive equipment and technical expertise.

**Fig 2:**
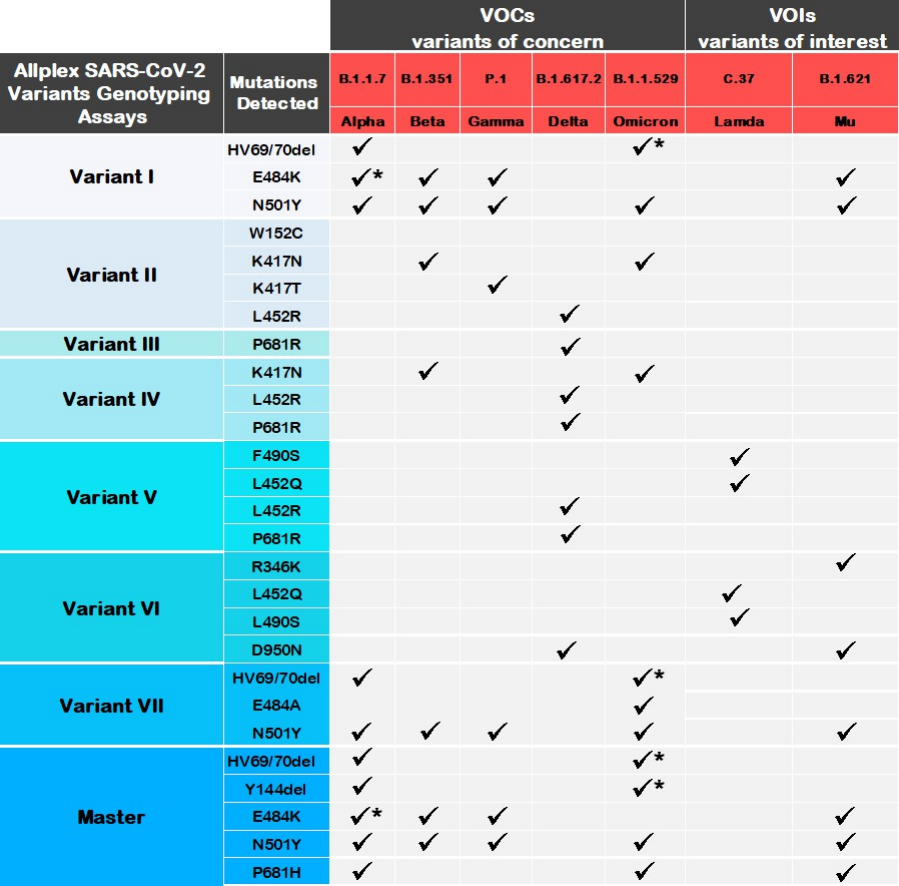
Current Allplex Seegene variant genotyping assays. Mutations detected by each assay and the corresponding variants are shown. Asterisks indicate variants for which the indicated mutation is not present in all sub-lineages.

The Seegene SARS-CoV-2 Variant assays (South Korea) detect mutations of different variants (Fig 2). We hypothesized that the Variant II assay, which was developed to detect K417N (Beta), K417T (Gamma), L452R (Delta) and W152C (Epsilon), would be a useful first line screening tool for monitoring and characterizing variants in resource-scarce and under-monitored settings. To test this, we evaluated feasibility of the Variants II assay in detecting prevalence of Beta and Delta between April and October 2021 in Limpopo, a resource-scarce and under-monitored province of South Africa, in the course of the third wave of infections during which the Beta and Delta variants circulated (Fig 1A).

## Materials and methods

### Sampling of SARS-CoV-2 specimens for variant typing

Ndlovu Research Centre and Laboratories (NRC) is situated in a rural community in Limpopo Province, South Africa. Since March 2020, NRC routinely receives SARS-CoV-2 clinical and community screening nasopharyngeal (NP) specimens for COVID-19 diagnostics and archives all SARS-CoV-2 positive samples tested on site. NP swabs are received in either eNAT® tubes with viral transport medium or as dry flocked swabs, which are subsequently resuspended in 2mL saline (0.9% NaCl). All positive swabs are stored at -80°C. Sampling period for the present analysis spanned from April to October 2021 during the third wave of SARS-CoV-2 infections in South Africa. For each month within this period, a random sampling of specimens with median Ct < 35 were selected from our biorepository for variant typing as described in Table 1. For samples with only two genes positive with Ct values below 35, the average of the two genes was used as the median. RNA extraction and a confirmatory SARS-CoV-2 RT-PCR was performed on samples prior to variant typing. All variant typing was performed using the Allplex Variants II RT-PCR assay.

**Table 1:** Number of samples received for COVID PCR diagnostics during the study period and percentage randomly selected for genotyping. For months with < 50 samples, all samples were selected for genotyping; for months with > 50 but less than < 500 samples, 15% were randomly sampled and for months with > 500 samples, 5% were randomly sampled.

Month	COVID-19 PCR Diagnostics Samples Received with Median Ct < 35	Number of Samples Selected for Genotyping	Percentage Sampled
April	12	12	100%
May	34	34	100%
June	400	60	15%
July	800	40	5%
August	110	17	15%
September	23	23	100%
October	1	1	100%
**Totals**	**1380**	**187**	

In total, 12, 34, 60, 40, 17, 23, 2 and 1 positive SARS-CoV-2 samples were variant typed for the months of April, May, June, July, August, September and October, respectively, for a total of 187 samples, comprising ~15% of all positives with median Ct < 35 processed at our laboratory during the sampling period. Metadata associated with all analysed samples from our biorepository are detailed in S1–S7 Tables No information that can be used to personally identify any individual, e.g., names, personal identification number, etc. were used or are included in the study. Ethics for the study was approved by the Wits Human Research Ethics Committee (HREC) (Ref. M200717 MED20-07-019), University of the Witwatersrand.

### Nucleic acid extraction

Total RNA extraction was performed using the SEEPREP32™ automated extraction system (Seegene Inc., South Korea) with the STARMag 96 ProPrep C extraction plates (Cat No: EX00017P, Seegene Inc., South Korea) according to manufacturer’s instructions with 200 μl of NP swab aliquot used as input. RNA was eluted in final volume of 100ul. After extraction, eluted RNA was kept at -20°C short-term (1–7 days) for RT-PCR and variant typing; and stored at −80°C long term.

### SARS-CoV-2 detection

SARS-CoV-2 RT-PCR was performed on extracted RNA using either the Allplex^TM^ 2019-nCoV-2 assay (Cat No: RP10243X) or the Allplex^TM^ SARS-CoV-2/FluA/FluB/RSV assay (Cat No: RV10259X) according to manufacturer’s instructions on the CFX96 DX Touch™ system (Bio-Rad Laboratories, Hercules, CA, USA).

Briefly, for the Allplex^TM^ 2019-nCoV-2 assay, RT-PCR was performed with the following conditions: reverse transcription at 50°C for 20 min, then 95°C for 15 min followed by 45 amplification steps of 15 seconds at 94°C and 30 seconds at 58°C. For the Allplex^TM^ SARS-CoV-2/FluA/FluB/RSV assay, reverse transcription was performed at 50°C for 20 min, then 95°C for 15 min followed by 3 cycles of initial PCR activation at 95°C for 10 seconds, 60°C for 40 seconds, and 72°C for 20 seconds, followed by 42 amplification cycles of 95°C for 10 seconds, 60°C for 15 seconds and 72°C for 10 seconds. Analytical performance of the two assays have been previously assessed in the literature and both assays have been shown to be comparable with no significant differences in performance [17]. For both assays, samples were interpreted as positive if one or more genes were detected with a Ct value ≤ 40. ` All positive samples detected by the Allplex^TM^ 2019-nCoV-2 assay were repeated on the Allplex^TM^ SARS-CoV-2/FluA/FluB/RSV assay for confirmation and only confirmed samples were used for SARS-CoV-2 Variant Typing PCR analysis.

### SARS-CoV-2 variant typing

Variant typing was performed on extracted RNA using the Allplex™ SARS-Cov-2 Variants II assay (Cat No: RV10305X, Seegene Inc., Republic of Korea) according to manufacturer’s instructions. Briefly, 20 μl of PCR reaction mixture per sample containing 15 μl of the Allplex™ SARS-Cov-2 Variants II master mix and 5 μl of eluted extracted RNA was loaded onto a CFX96 Touch™ system (Bio-Rad Laboratories, Hercules, CA, USA) and RT-PCR performed with the following conditions: reverse transcription at 50°C for 20 minutes, then 95°C for 15 minutes, followed by 3 cycles of PCR initial activation at 95°C for 10 seconds, 60°C for 40 seconds, and 72°C for 20 seconds, and lastly 42 amplification steps of 10 seconds at 95°C, 15 seconds at 60°C and 10 seconds at 72°C. Fluorescent signal is detected in the FAM (L452R mutation), HEX (W152C mutation), Cal Red 610 (K417T mutation), Quasar 705 (K417N mutation), and Quasar 670 (internal control) channels. Positive and negative controls were included in each run and the SARS-CoV-2 Viewer V1 Trial Variants II software (Seegene Inc., Republic of Korea) was used for automated analysis and interpretation of results.

### SARS-CoV-2 whole genome sequencing

Whole genome sequencing (WGS) was performed using either Illumina MiSeq (Illumina Inc., USA), PacBio Sequel IIe (Pacific Biosciences Inc., USA) or Genexus Ion Torrent (Thermo Scientific, USA) platforms. SARS-CoV-2 WGS with Illumina MiSeq was performed on libraries prepared using Nextera DNA Flex Library kit (Illumina) according to the protocol described in [18]. For PacBio, the manufacturer’s Sequel IIe WGS workflow was used. Briefly, fourteen overlapping 2.5 kb amplicons based on primers designed by John-Eden Sebastian, University Sydney, Australia, tiled across the 29 kb genome was used. Finally, for Genexus sequencing, Thermo Fisher manufacturer’s instructions for the Ion AmpliSeq™ SARS‑CoV‑2 Research Panel workflow was used.

### Statistical and data analysis

Descriptive statistics for continuous variable Ct values were calculated with the median and standard deviation. Diagnostic performance was assessed by routine metrics of diagnostic accuracy (sensitivity, specificity, positive predictive value, negative predictive value), and when applicable was reported with 95% confidence intervals (95% CI). All analysis and visualization was performed with Microsoft Excel, Google Sheets and R version 4.0.3 [19].

### Data availability

Sequencing methodologies are provided in this material and method sections. Associated demographic information for all genotyped samples are included in S1–S7 Tables.

## Results

To evaluate the feasibility of the Allplex^TM^ Variants II genotyping assay, we assessed (a) specificity for identifying the Beta and Delta variants, (b) accuracy in identifying the Beta and Delta variants compared to whole genome sequencing methods, (c) linearity across a range of SARS-CoV-2 S-gene cycle threshold (Ct) values, (d) reproducibility across different operators and (e) repeatability within the same operator.

Specificity was evaluated by testing the Variants II assay for cross-reactivity to 160 specimens comprising non-SARS-CoV-2 samples and SARS-CoV-2 positive samples obtained in 2020, before emergence of Beta and Delta variants. Briefly, the following samples were tested for cross reactivity: 32 influenza-A H1-pdm09 samples, 2 influenza-A H3 samples, 11 influenza B samples, 2 *Haemophilus influenzae* samples, 1 respiratory syncytial virus (RSV) sample, 20 SARS-CoV-2 samples obtained prior to emergence of Beta and Delta and 92 SARS-CoV-2 negative samples obtained during routine COVID-19 PCR testing. No cross-reactivity with Variant II PCR was observed for any of the above samples, demonstrating high specificity of the assay (S1A–S1C Table).

Accuracy of the Variants II assay was assessed by performing variant typing on 38 SARS-CoV-2 samples that had been previously sequenced by different next generation whole genome sequencing (NG-WGS) platforms. Of the 38 samples sequenced, 22 were sequenced by Illumina MiSeq; 10 by Ion Torrent, and 6 by PacBio NG-WGS. Of the 22 samples sequenced by Illumina, 6 and 16 were identified as Beta and Delta, respectively, and were also variant typed as Beta and Delta by the Variants II assay. Of the 10 and 6 samples sequenced by Ion Torrent and PacBio, respectively, all were identified as Delta and all 16 samples were also typed as Delta by the Variant II assay (S2 Table). In summary, variant typing by the Variants II Assay on sequenced samples yielded identical results when compared to NG-WGS platforms tested resulting in a 100% positive agreement for evaluated samples (Table 2).

**Table 2:** Accuracy of Variant II assay in detecting variants confirmed by whole genome sequencing. A total of 38 sequenced confirmed variants were analysed by the Variants II assay. Of the 38 sequenced samples, 22 were sequenced by Illumina MiSeq, 10 by IonTorrent Genexus and 6 by PacBio Sequel IIe (see Materials and methods).

Variant II Assay	Whole Genome Next Generation Sequencing								Totals
	Illumina				IonTorrent		PacBio		
	Beta		Delta		Delta		Delta		
	Detect	ND	Detect	ND	Detect	ND	Detect	ND	
**Detect**	6	0	16	0	10	0	6	0	38
**Not Detected (ND)**	0	0	0	0	0	0	0	0	0
									38
								**PPA =**	100%

**Fig 3:**
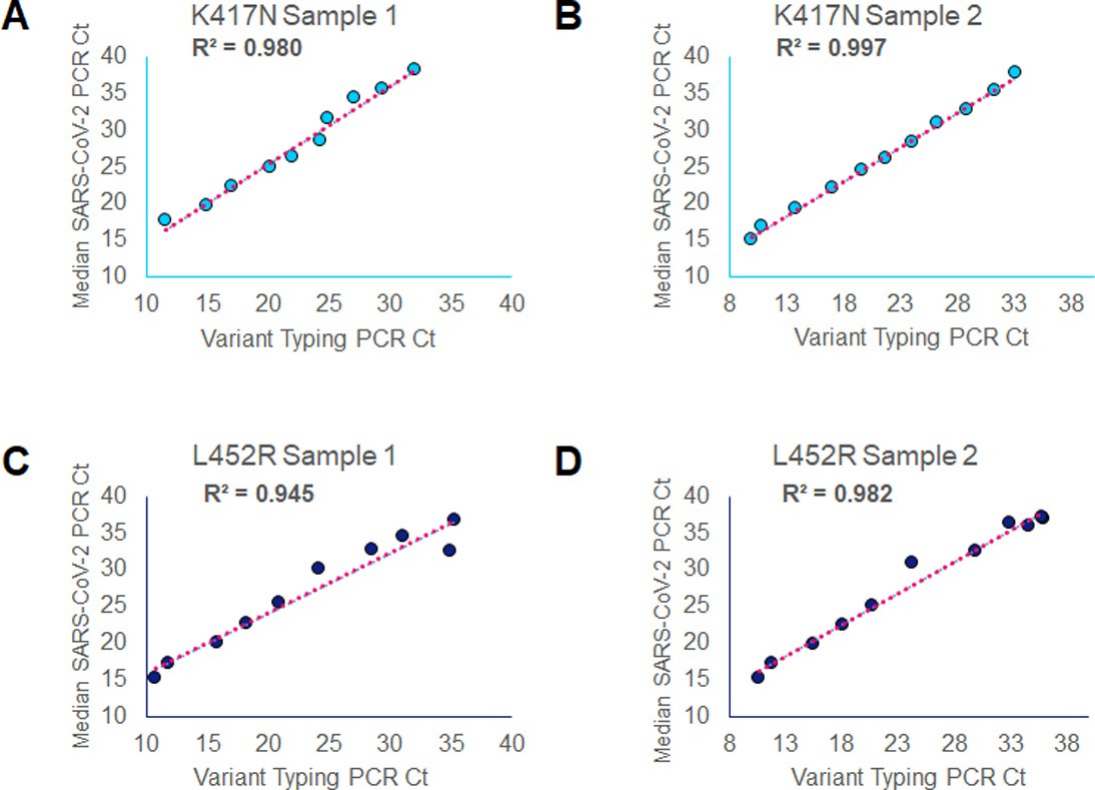
Correlation between variant typing and median SARS-CoV-2 PCR Ct values. Variant II PCR Cts (x-axes) and SARS-CoV-2 PCR median Cts (y-axes) of 2 different K417N (A and B) and 2 different L452R (C and D) 5-fold serial diluted samples.

To evaluate linearity of the assay, 2 Beta and 2 Delta variant samples with an average SARS-CoV-2 median Ct value of 10.75, SD ±0.56 were diluted 5-fold until all three genes (S, RdRp, and N) were undetectable by SARS-CoV-2 RT-PCR. Each dilution excluding the undetectable sample were then tested by Variant II assay to determine correlation between the Variant RT-PCR Ct value and the SARS-CoV-2 RT-PCR Ct values. Pearson correlations between SARS-CoV-2 median Ct and variant typing Ct values for the four samples analysed were 0.980, 0.997, 0.945 and 0.982, yielding an average correlation of 0.976 (SD ± 0.019) (Fig 3 and S3 Table). The average of the median SARS-CoV-2 Ct values of the 2 Beta and 2 Delta dilutions before the limit of detection was reached was 34.1 (SD ± 1.57), suggesting that samples with median SARS-CoV-2 Ct values > 35 are not likely to be variant typed successfully on the Variants II assay.

**Fig 4:**
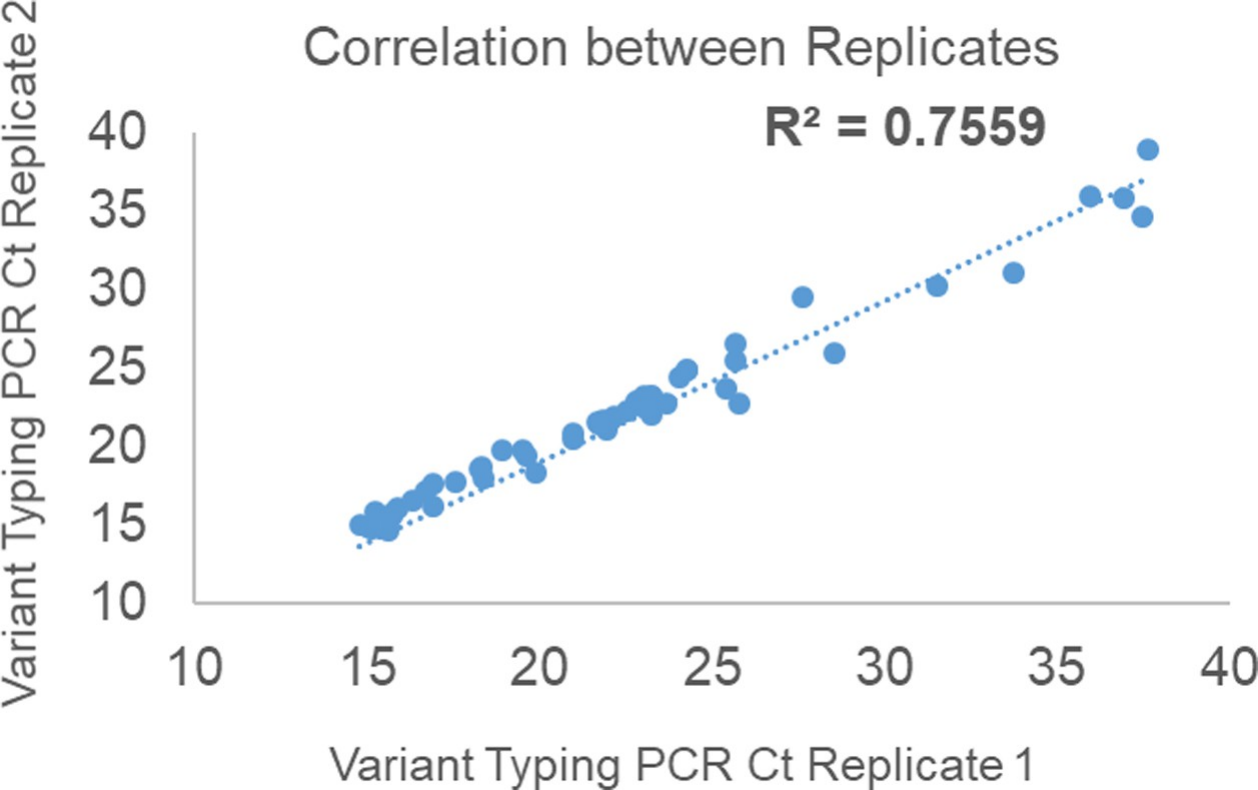
Correlation between replicate Ct values of 53 samples analyzed by Variant II PCR.

To evaluate reproducibility, purified RNA of 5 Delta variant samples and positive and negative controls were analysed on the Variant II assay by two independent operators on the same day. Results were identical across the two operators with near identical Ct values (average SD: ± 0.13) (S4 Table). Finally, to evaluate assay repeatability, purified RNA of 39 SARS-CoV-2 positive samples and 16 positive controls samples for a total of 55 samples were analysed in duplicate on the Variant II assay by the same operator on the same day. Of the 55 replicates 2 samples were discordant (2 invalids in one replicate but not the other) while the rest yielded identical results across the two replicates (96.4% repeatability concordance). Correlation of Ct values between the two replicates was 0.76 with average SD of ± 0.33 between replicates (Fig 4 and S5 Table).

**Fig 5:**
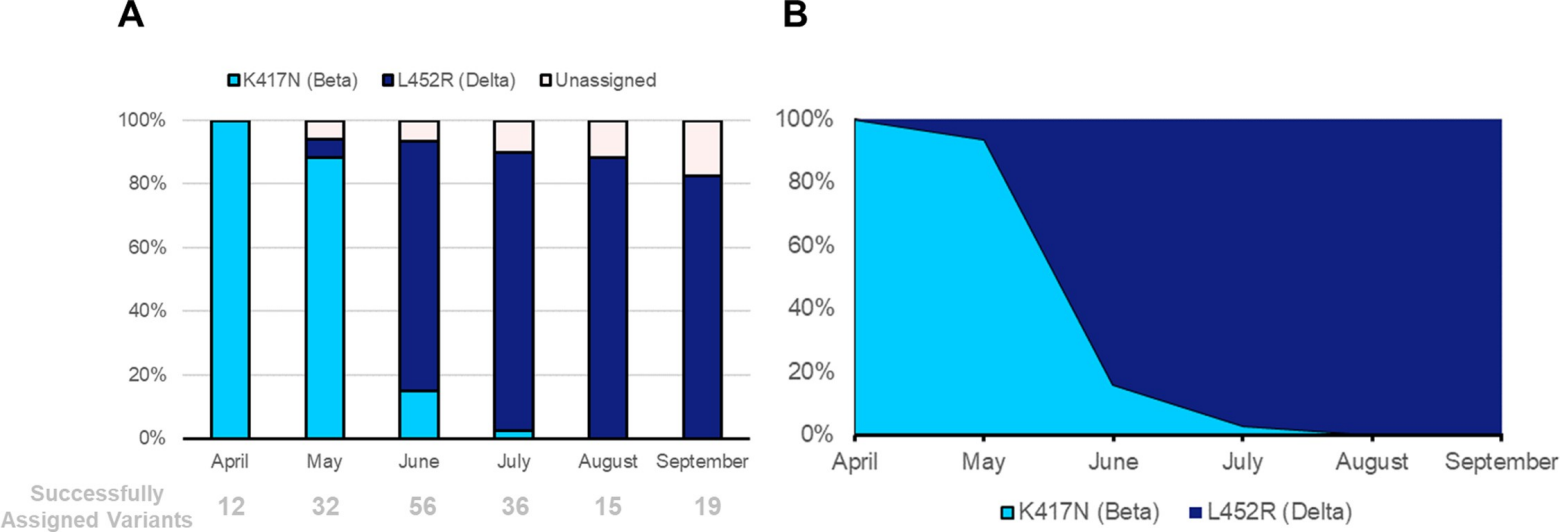
Variant distribution for Limpopo specimens sampled between April and September 2021. Graphs show the rapid displacement of the Beta (cyan) variant by the Delta (dark blue) variant. (A) Variant distribution for all samples analyzed and (B) Distribution of only the samples that were successfully assigned a variant.

After establishing specificity, accuracy, sensitivity, reproducibility and repeatability of the Variant II assay in detecting and differentiating between Beta and Delta variants, we implemented the assay to characterize circulating variants between April and October 2021 in the Limpopo Province of South Africa and to gain insight into time of introduction of the Delta variant in the province. Overall, 187 samples collected between April and October 2021 were genotyped by the Variant II assay. Of the genotyped samples, 171 were successfully assigned a variant yielding a variant typing success rate of 91.4% (Table 3). Fig 5A and 5B, respectively, illustrate the proportion of genotyped results and the distribution of successfully identified variants from April through September 2021. For the month of April, all samples were successfully typed by the Variants II assay and only the Beta variant was detected. In the month of May, 30 of the 34 genotyped samples were Beta variants, 2 were Delta and 2 samples failed to be assigned a variant (Beta variant 88%, Delta variant 6%, unassigned, 6%; Fig 5A). This indicates that the Delta variant was present in Limpopo by the May 2021. In June, a drastic shift from Beta to Delta variants was observed: 9 of the 59 samples were Beta variants and 47 were Delta variants and 4 samples failed to be assigned a variant (Beta variant 15%, Delta variant 78%, unassigned 7%, Fig 5A). For July, only 1 of the 38 genotyped samples was a Beta variant; 35/38 were Delta, and 4 were unassigned (Beta variant 3%, Delta variant 88%, unassigned 10%, Fig 5A). From August till October, only Delta variants were identified amongst successfully genotyped samples. Due to the fact that only 1 SARS-CoV-2 positive sample with median Ct value < 35 was received for October, variant distribution for that month was not performed as the sample size was too limited (Table 3 and Fig 5). Information for all samples used in this study and associated metadata are provided in S1–S7 Tables.

**Table 3:** Number of samples genotyped per month and amount of samples successfully genotyped.

Month	Samples Genotyped	Assigned Variants	Unassigned Variants
April	12	12	0
May	34	32	2
June	60	56	4
July	40	36	4
August	17	15	2
September	23	19	4
October	1	1	0
**Totals**	**187**	**171**	**16**

To further investigate samples that were not successfully genotyped by the Variant II assay, we performed whole genome sequencing using the Ion Torrent platform on 15 of the unassigned variant samples. Of the 15 unassigned samples, whole genome sequencing identified 7 Delta variants, while the rest were either variants not detectable by the Variants II assay or that were not able to be sequenced. Thus, 8/15 of samples that could not be genotyped by PCR variant typing in our analysis were neither Beta nor Delta variants (negative predictive value 53.3% (95% CI 0.52–0.54). Taken together, our results showed acceptable specificity, accuracy, linearity, reproducibility, and repeatability of the Variants II assay in identifying Beta and Delta variants and shed important insight into variant distribution and the time of introduction of the Delta variant in the Limpopo Province of South Africa between April and October 2021.

## Discussion

Whole genome sequencing (WGS) remains the gold standard for genomic surveillance; however, it is cost-, time-, and labour-intensive, and requires well-representative sampling of specimens to provide an accurate picture of variant distribution in a population. In contrast, multiplex PCR-based genotyping offers a rapid and cost-effective approach to screen and identify variants and can complement existing WGS strategies. In this study we assessed the feasibility of the Seegene Allplex^TM^ Variants II genotyping assay to identify VOCs in an under-monitored and resource-scarce South African setting.

The assay accurately identified Beta and Delta variants in sequence-confirmed SARS-CoV-2 clinical samples and provided key insight into the emergence and expansion of the Delta variant in the Limpopo Province. Our findings show that by late May 2021 the Delta variant was already circulating in Limpopo, by June it had begun to outcompete the Beta variant, and by July 2021 had completely replaced Beta to become the dominant variant. Significantly, our results are highly consistent with WGS Limpopo data reported by the South African Network for Genomic Surveillance (NGS-SA) as well other reports covering the time period we analysed [15, 20]. Furthermore, our results are consistent with similar studies that have recently evaluated Seegene Variant assays [21, 22]. This observation reinforces the predictive value of commercial multiplex PCR genotyping assays in epidemiological surveillance and highlights the diagnostic utility this approach can play in supporting WGS in resource-scarce and under-represented settings, as has been shown in other studies [23–29]. Additionally, this strategy is rapid, reproducible, and technically easy. Table 4 briefly summarizes the strengths and weaknesses of multiplex genotyping PCR compared to WGS, including comparisons of time to results, cost of kits, training, and infrastructure needed.

**Table 4:** Comparing strengths and weaknesses of RT-PCR variant genotyping and whole genome sequencing. Time to results (turnaround time), cost, technical expertise are contrasted between the two methods.

Assay	Strengths and Weaknesses	
	Advantages	Disadvantages
**RT-PCR Variant Genotyping**	Quick (~2hr turnaround time)	Low—medium throughput
	Cost effective (~$5.00/sample)	Only detects key mutations of known variants
	Technically simple	Does not directly detect novel variants
	No complex software required for data analysis	
**Next Generation Whole Genome Sequencing (NGS)**	Covers entire SARS-CoV-2 genome	High turnaround time (~24hrs—several days)
	Detects all mutations of known and novel variants	Expensive (~$100-$300/sample)
	Highly sensitive	Requires high technical skills
	High throughput	Complex bioinformatics pipelines required for data analysis

We observed that samples with high Ct values (median gene Ct >35) are not readily genotyped, therefore careful selection of samples is necessary for PCR variant typing. Of note, the Ct value threshold at which PCR variant typing becomes infeasible correlates with the Ct threshold value at which SARS-CoV-2 WGS becomes difficult [30–32].

**Fig 6:**
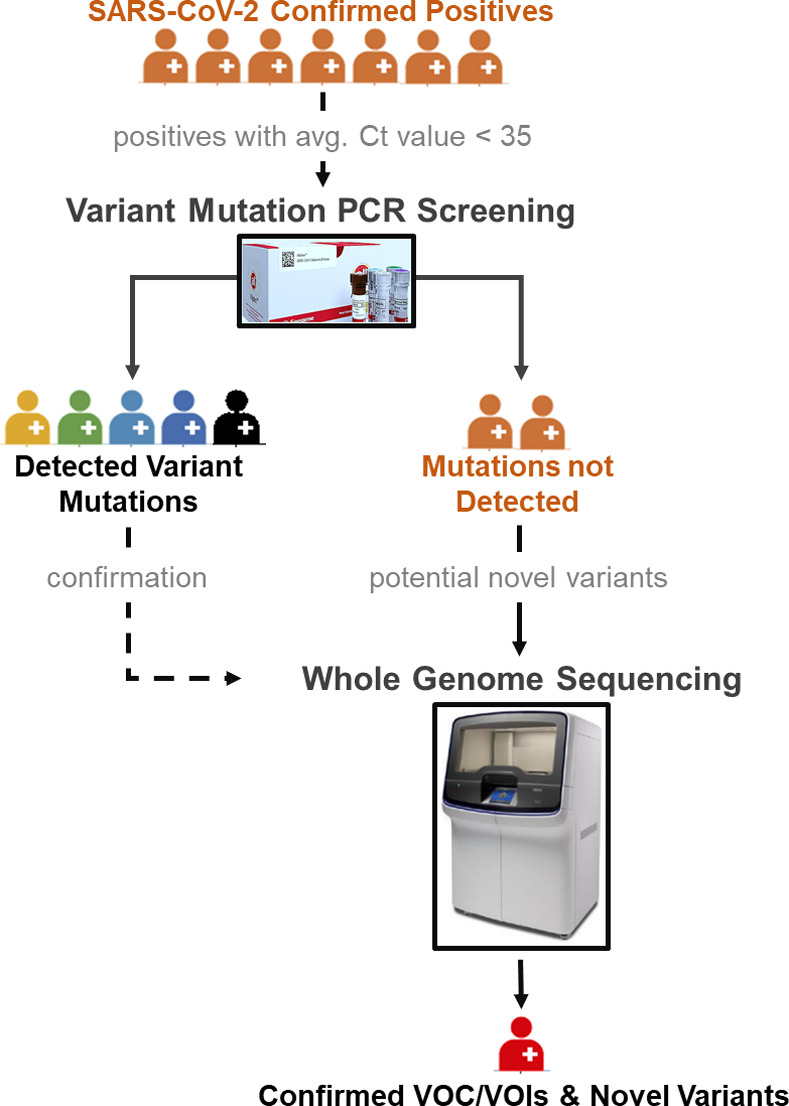
Proposed algorithm for SARS-CoV-2 variants detection and characterization using multiplex genotyping PCR and genomic sequencing assays. Confirmed SARS-CoV-2 positives are first screened by Variant genotyping PCR. Samples with detected variant mutations can be confirmed by whole genome sequencing for precise variant classification as needed. Samples for which variant mutations are not detected should be analysed by whole genome sequencing to assess possibility of emerging novel variants.

Commercial multiplex PCR genotyping kits can be combined to maximize variant detection capacity and identify novel variants. There are a number of variant PCR assays which, when combined, allow for detection of multiple SAR-CoV-2 variants [21, 22, 28, 29, 33, 34]. Fig 2 summarizes variants detectable by currently available Allplex^TM^ Variant assays. Variant PCR assays can also provide evidence of recombination or mixed populations if unique mutations of multiple variants are observed in a single genotyped sample. Additionally, samples with low S gene Ct values that fail to be assigned a variant by genotyping could be harbouring novel mutations and can be further investigated by WGS. Fig 6 outlines a potential algorithm for complementing genotyping assays with WGS for variant identification and screening for novel mutations.

## Conclusions

The usage, implementation, and fast adoption of well-validated commercial genotyping assays are needed to expand ongoing surveillance efforts. The strategy outlined in this report offers a powerful method of decentralizing surveillance activities and paves the way for dramatically scaling up national surveillance efforts, rural capacity building, and enhanced coverage of neglected and under-represented provinces in South Africa and other resource-limited settings.

The present study has several limitations, the main one being that genotyping PCR assays cannot directly identify novel variants since they are designed to identify variants whose mutations are already known. Another limitation of this method arises when multiple variants share the same mutation(s) that is detected by a given genotyping assay, in which case further analyses to conclusively classify the variant are needed. In the case of the Variants II assay used in this study, the K417N mutation is used as a proxy marker for the Beta variant but is also present in Omicron. Detection of the K417N mutation by the Variant II assay is, therefore, indicative of either Beta or Omicron and additional analyses are needed for conclusive variant typing. Finally, as has been pointed in other studies, variant genotyping requires careful selection of samples as viral copy number and sample integrity are important prerequisites for successful genotyping [29, 35]. Upon completion of our analyses, the Omicron variant was identified in South Africa and subsequently took over as the dominant variant. However, since our analysis was already concluded prior to emergence of Omicron, genotyping of Omicron samples was not included in this study.

## Supporting information

S1 Table A. Cross-reactivity specificity testing of the Variants II assay with 48 non-SARS-CoV-2 respiratory pathogens. B. Cross-reactivity specificity testing of the Variants II assay with 20 non- Beta and Delta SARS-CoV-2 positive samples obtained in 2020, before emergence of those variants. C. Cross-reactivity specificity testing of the Variants II assay with 92 SARS-CoV-2 negative samples.
(XLSX)

S2 Table Accuracy of the Variants II assay as assessed by comparison to whole genome sequencing. 38 SARS-CoV-2 samples that had been previously sequenced by either Illumina MiSeq; Ion Torrent, or PacBio methods were genotyped by the Variant II assay.
(XLSX)

S3 Table Linearity measurements for the Variants II assay. 2 Beta (K417N) and 2 Delta (L452R) variant samples were diluted 5-fold until not detectable by SARS-CoV-2 RT-PCR and analysed by Variants II assay.
(XLSX)

S4 Table Reproducibility assessment for Variants II. Two operators analysed 5 samples and a positive and negative control in parallel.
(XLSX)

S5 Table Repeatability assessment for the Variant II assay. 55 samples were analysed in duplicate by the same operator.
(XLSX)

S6 Table Variants II results for analysed samples obtained between April and October 2021. (XLSX)

S7 Table Whole genome sequencing follow-up of samples in which no variant mutation was detected by Variant II assay. (XLSX)
